# hiPSC-derived cardiomyocytes from Brugada Syndrome patients without identified mutations do not exhibit clear cellular electrophysiological abnormalities

**DOI:** 10.1038/srep30967

**Published:** 2016-08-03

**Authors:** Christiaan C. Veerman, Isabella Mengarelli, Kaomei Guan, Michael Stauske, Julien Barc, Hanno L. Tan, Arthur A. M. Wilde, Arie O. Verkerk, Connie R. Bezzina

**Affiliations:** 1 Heart Centre, Department of Experimental and Clinical Cardiology, Academic Medical Center, University of Amsterdam, Amsterdam, The Netherlands; 2 Department of Cardiology and Pneumonology, Georg-August-University Göttingen, Göttingen, Germany; 3 l’institut du thorax, INSERM, CNRS, Université de Nantes, Nantes, France; 4 Department of Anatomy, Embryology and Physiology, Academic Medical Center, University of Amsterdam, Amsterdam, The Netherlands

## Abstract

Brugada syndrome (BrS) is a rare cardiac rhythm disorder associated with sudden cardiac death. Mutations in the sodium channel gene *SCN5A* are found in ~20% of cases while mutations in other genes collectively account for <5%. In the remaining patients the genetic defect and the underlying pathogenic mechanism remain obscure. To provide insight into the mechanism of BrS in individuals without identified mutations, we here studied electrophysiological properties of cardiomyocytes (CMs) generated from human induced pluripotent stem cells (hiPSCs) from 3 BrS patients who tested negative for mutations in the known BrS-associated genes. Patch clamp studies revealed no differences in sodium current (*I*_Na_) in hiPSC-CMs from the 3 BrS patients compared to 2 unrelated controls. Moreover, action potential upstroke velocity (V_max_), reflecting *I*_Na_, was not different between hiPSC-CMs from the BrS patients and the controls. hiPSC-CMs harboring the BrS-associated *SCN5A*-1795insD mutation exhibited a reduction in both *I*_Na_ and V_max_, demonstrating our ability to detect reduced sodium channel function. hiPSC-CMs from one of the BrS lines demonstrated a mildly reduced action potential duration, however, the transient outward potassium current (*I*_to_) and the L-type calcium current (*I*_Ca,L_), both implicated in BrS, were not different compared to the controls. Our findings indicate that ion channel dysfunction, in particular in the cardiac sodium channel, may not be a prerequisite for BrS.

Brugada syndrome (BrS) is a rare inheritable cardiac disorder characterized by ST-segment elevation and a negative T-wave in leads V1–V3 of the ECG and an increased risk of sudden cardiac death from ventricular fibrillation (VF)^1^,^2^. In spite of intense research by means of clinical, experimental and genetic studies, the basis for these ECG abnormalities and the mechanism underlying VF in this disorder are incompletely understood.

The *SCN5A* gene, mutated in ~20% of BrS probands^3^, is thus far the only gene that has unequivocally been associated with the disorder^4^. *SCN5A* encodes the major sodium channel isoform in heart (Na_v_ 1.5), which underlies the cardiac Na^+^ -current (*I*_Na_) responsible for rapid cardiomyocyte depolarization and as such is a key player in cardiac conduction^5^. BrS causing mutations in *SCN5A* result in loss of *I*_*Na*_ and are thought to contribute to the disorder through the slowing of conduction, a mechanism that is believed to be central in the pathogenesis of the disorder^6^. Mutations in other genes have been found in a small percentage of BrS patients; these encode proteins that modulate *I*_*N*a_ (interacting proteins or beta-subunits) or lead to the loss- or gain-of-function of the L-type Ca^2+^ -current (*I*_Ca,L_) or the outward K^+^ -currents (*I*_KATP_ and *I*_to_), respectively^2^. In the vast majority of BrS patients, however, no causal mutation is identified and studies investigating comprehensive lists of genes encoding candidate ion channel genes and their modulatory subunits have not uncovered strong associations with the disorder^7^. In these patients it remains unclear whether the disorder is caused, at least in part, by an ion channel defect or whether the disorder is caused to a large part by other factors. Such other factors may be extrinsic to the cardiomyocyte, such as the deposition of fibrosis, or may involve defects of cardiomyocyte electrical coupling through gap junctions, the relevance of which is increasingly recognized^8^,^9^,^10^.

The possibility to generate cardiomyocytes (CMs) from human induced pluripotent stem cells (hiPSCs, hiPSC-CMs) from patient material provides the opportunity to study the electrophysiological characteristics of patient-specific cardiomyocytes, with the possibility of gleaning insights into disease mechanisms^11^. In this study we generated hiPSC-CMs from three unrelated BrS patients that were negative for coding region mutations in BrS-associated genes and compared their electrophysiological properties to hiPSC-CMs from two unrelated control individuals. We demonstrate that hiPSC-CMs from the three BrS patients do not exhibit Na^+^ channel dysfunction and that action potential (AP) characteristics do not point towards a clear cellular electrophysiological defect. hiPSC-CMs from one of the BrS lines demonstrated a mild reduction in AP duration, however, neither *I*_to_ nor *I*_Ca,L_ differed significantly from hiPSC-CMs from the two controls. This supports the hypothesis that ion channel dysfunction, in particular in the cardiac sodium channel, may not be a prerequisite for BrS.

## Results

### Patient characteristics

**Figure 1:**
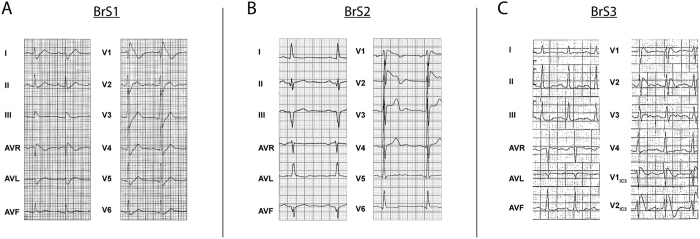
Electrocardiogram (25 mm/s, 10 mm/mv) of BrS1 (**A**), BrS2 (**B**) and BrS3 (**C**). Type 1 BrS signs are visible in leads V1–V2 (**A,B**) or V1_IC3_–V2_IC3_ (**C**) (leads positioned the 3rd intercostal space cranial from V1 and V2).

BrS was diagnosed following the 2005 consensus diagnostic criteria^12^. The 1^st^ patient we included in this study (BrS1) was a 42-year-old male who presented at night with an out-of-hospital cardiac arrest based on VF. His medical history included unexplained syncope 2 years earlier, but was otherwise unremarkable. He took no medications and there was no obvious cause for VF. He had a type 1 BrS ECG (>2 mm coved-type ST-elevation and a negative T-wave) in lead V1 (Fig. 1A). QRS-duration was increased (120 ms) while the PR- and QTc-intervals were within the normal range. QTc intervals were determined in lead II from three consecutive complexes and corrected for heart rate according to Bazzett’s formula^13^. Magnetic resonance imaging showed no structural abnormalities and coronary angiography demonstrated no coronary artery disease. His first- and second-degree relatives did not show baseline ECG abnormalities, however provocation with ajmaline evoked a type 1 BrS ECG in 3 out of 6 individuals tested. No history of sudden cardiac death was present in the patient’s family.

The 2^nd^ patient (BrS2) was a 67-year-old male with a history of syncope who presented with a type 1 BrS ECG during a hospital admission for an inferior myocardial infarction (Fig. 1B). His ECG showed a prolonged PR-interval (230 ms) with a normal QRS-duration and QTc-interval. Moreover, the patient had episodes of paroxysmal atrial fibrillation. During electrophysiological investigation ventricular arrhythmia could be evoked, after which an ICD was implanted. Apart from the proband, no relatives were identified with a type 1 BrS ECG at baseline, while flecainide provocation tests in 15 first- and second-degree relatives revealed 6 individuals fulfilling the ECG criteria of BrS. In the patient’s family, sudden cardiac death was reported in several members.

The 3^rd^ patient (BrS3) was a 24-year-old female who presented at the outpatient clinic because of episodes of syncope. She had a type 1 BrS ECG seen in ECG leads placed over the 3^rd^ intercostal space cranially from V1 and V2 (Fig. 1C). Additional electrophysiological investigation revealed inducible non-sustained ventricular tachycardias in the right-ventricular outflow tract, after which an ICD was placed. Conduction parameters revealed PQ-interval prolongation (220 ms), with a normal QRS-duration and QTc interval. Screening of family members by ajmaline provocation revealed 5 individuals fulfilling the BrS ECG diagnostic criteria among 12 individuals tested in whom no ECG abnormalities were identified at baseline. No history of sudden cardiac death was reported in the family of the patient.

Genetic screening of the coding region of *SCN5A* did not uncover any novel or rare variants (MAF <1%) in the three patients. Moreover, BrS1 and BrS2 were negative for such variants in all other BrS-associated genes. BrS3 carried a rare variant in the *CACNA1C* gene (present in 5 of 120140 alleles in the ExAC database). However, this variant, which is present at position −7 of intron 19 (numbering according to Ensembl transcript ENST00000399634), does not appear to entail a nucleotide change that negatively affects splicing (pyrimidine to pyrimidine change in the polypyrimidine tract), and analysis of the wild-type and variant sequence surrounding the 3′ splice site of intron 19 using three different splice site prediction programs^14^,^15^,^16^ did not uncover any splicing motif alteration. This led us to conclude that this variant has probably no impact on splicing.

### Generation of hiPSCs and hiPSC-CMs

Dermal fibroblasts from the three patients were reprogrammed into hiPSCs through lentiviral transduction of the transcription factors *OCT4*, *KLF4*, *SOX2*, *c-MYC*; the three lines thus generated are hereafter annotated as iBrs1-3 for patients BrS1, BrS2 and BrS3 respectively. The hiPSC lines showed alkaline phosphatase activity and stained positive for the pluripotency markers OCT4, SOX2, LIN28, SSEA-4 and Tra-1-60. (Supplementary Fig. 1A–C). Moreover, spontaneous differentiation resulted in the formation of cell types from the three germ layers, ectoderm, mesoderm and endoderm, as evidenced by immunostaining for the proteins β-III-Tubulin, α-SMA and AFP, respectively (Supplementary Fig. 2). The karyotypes of iBrs1-3 were normal (Supplementary Fig. 3). Differentiation of hiPSCs into CMs was confirmed by positive immunostainings of the cardiac markers NKX2.5 and cTNT (Supplementary Fig. 4)

### Electrophysiological characteristics of hiPSC-CMs from Brugada Syndrome patients

Patch-clamp electrophysiological studies were conducted in hiPSC-CMs generated from the three BrS patients (iBrS1, iBrS2 and iBrS3) and in hiPSC-CMs from the two controls (iCtrl1 and iCtrl2). In addition, hiPSC-CMs derived from a patient with the 1795insD mutation in *SCN5A* (iSCN5A)^17^ served as a positive control to demonstrate our ability to detect reduced *I*_Na_ function, and its consequences on the AP, in our experimental system.

**Figure 2:**
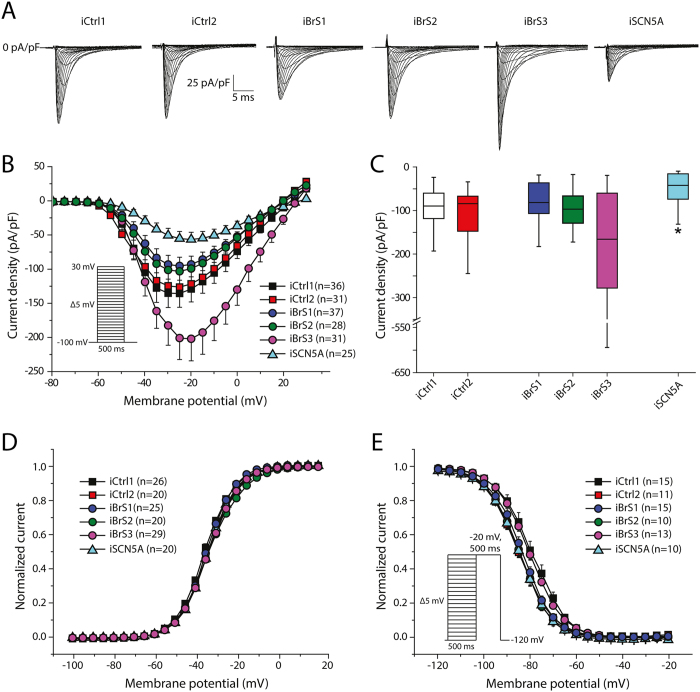
Sodium current (*I*_Na_) characterization in hiPSC-CMs from iCtrl1, iCtrl2, the three BrS patients iBrS1 to iBrS3 and iSCN5A. (**A**) Representative traces of *I*_Na_ as determined by the voltage clamp protocol shown in the inset of (**B**). (**B**) Current-voltage (I-V) relationships of peak *I*_Na_ (mean ± SEM). (**C**) Boxplots depicting *I*_Na_ densities (median, boxes represent interquartile range, whiskers 95% interval), determined at −20 mV. *indicates significance compared to iCtrl1 and iCtrl2 (p < 0.05; Kruskal-Wallis test, followed by pairwise comparisons). (**D**) Voltage dependence of activation. (**E**) Voltage dependence of inactivation. The inset represents the voltage clamp protocol that was applied.

Figure 2A shows representative traces of *I*_Na_ from all groups. Average peak *I*_Na_ of iBrS1, iBrS2 and iBrS3 hiPSC-CMs did not differ significantly from the two controls (Fig. 2B,C). In contrast, iSCN5A displayed a marked reduction in *I*_Na_ density (p < 0.01 versus iCtrl1 and iCtrl2; Fig. 2B,C). Voltage dependence of *I*_Na_ activation did not differ significantly between the groups (Fig. 2D and Table 1). Voltage dependence of inactivation of iBrS1, iBrS2 and iSCN5A did not differ significantly from iCtrl2, while iBrS3 displayed similar values as iCtrl1 (Fig. 2E and Table 1). The voltage dependence of inactivation of iCtrl1 and iCtrl2 differed significantly between each other, with iCtrl1 displaying a slight positive shift in V_1/2_ of inactivation (p < 0.05; (Fig. 2E and Table 1). The time constants of current inactivation, determined at a test potential of −30 mV, were not different in iBrS1, iBrS2 and iBrS3 compared with the two controls. hiPSC-CMs from iSCN5A showed slower time dependence of current inactivation, in accordance with an increased late *I*_Na_ induced by the 1795insD mutation (Table 1) (p < 0.01).

**Figure 3:**
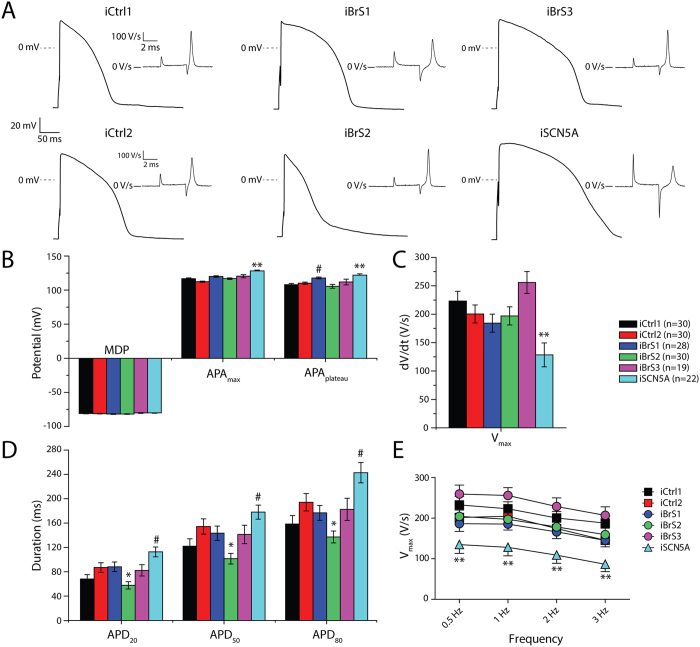
AP characteristics as measured after addition of *in silico I*_K1_ in hiPSC-CMs from iCtrl1, iCtrl2, the three BrS patients iBrS1 to iBrS3 and iSCN5A. (**A**) Typical examples of APs measured at 1 Hz. Inset show the first derivatives displaying AP upstroke velocity. (**B**) Averages of maximum diastolic potential (MDP), maximum AP amplitude (APA_max_) and amplitude of the plateau (APA_plateau_), measured after 10 ms from the onset of the upstroke. (**C**) Maximal upstroke velocity (V_max_) at 1 Hz. V_max_ in hiPSC-CMs from iBrS1, iBrS2 and iBrS3 is not significantly different from iCtrl1 and iCtrl2, in contrast to iSCN5A, which shows a marked reduction (p < 0.05; ANOVA, followed by Bonferroni posthoc tests). (**D**) Action potential duration (APD) at 20, 50 and 80% of repolarization (APD_20_, APD_50_ and APD_80_, respectively). APD is reduced in iBrS2 compared to iCtrl2 (p < 0.05), while iSCN5A exhibits an increase in APD compared to iCtrl1 (p < 0.05; ANOVA, followed by Bonferroni posthoc tests). (**E**) Rate dependency of V_max_ at frequencies of 0.5 to 3 Hz. No additional differences are exposed at higher or lower frequencies. **p < 0.05 vs iCtrl1 and iCtrl2. ^#^p < 0.05 vs iCtrl1 *p < 0.05 vs iCtrl2.

Next, we measured APs in single hiPSC-CMs. As explained in detail in the Methods section this was done after *in silico* addition of *I*_K1_ (see Supplementary Fig. 5 for an example of computed *I*_K1_). Fig. 3A shows typical APs stimulated at 1 Hz. Maximal AP upstroke velocity (V_max_), reflecting *I*_Na_ function^18^, was not significantly different between iCtrl1, iCtrl2, iBrS1, iBrS2 and iBrS3 (Fig. 3B). In contrast, iSCN5A demonstrated markedly reduced V_max_ (p < 0.05 versus iCtrl1 and iCtrl2), consistent with the reduced *I*_Na_ density (Fig. 2B). Maximal diastolic potential (MDP), AP plateau amplitude (APA_plateau_) and action potential duration (APD) at 20, 50 and 80% of repolarization (APD_20_, APD_50_ and APD_80_, respectively) were not statistically significantly different between the various BrS groups and the controls, with exception of iBrS2, which displayed shorter APD compared to one of the two controls (iCtrl2, p < 0.05) (Fig. 3C). In addition, iSCN5A exhibited a marked increase in APD, AP maximal amplitude (APA_max_) and APA_plateau_, in line with an increased late *I*_Na_ that is induced by the mutation, as demonstrated previously^17^. Of note, all AP parameters in iCtrl1 and iCtrl2 were not different between each other (p > 0.05 for all parameters). No additional differences in V_max_, APD and APA_plateau_ were uncovered upon stimulation at frequencies of 0.5–3 Hz (Fig. 3E and Supplementary Fig. 6).

**Figure 4:**
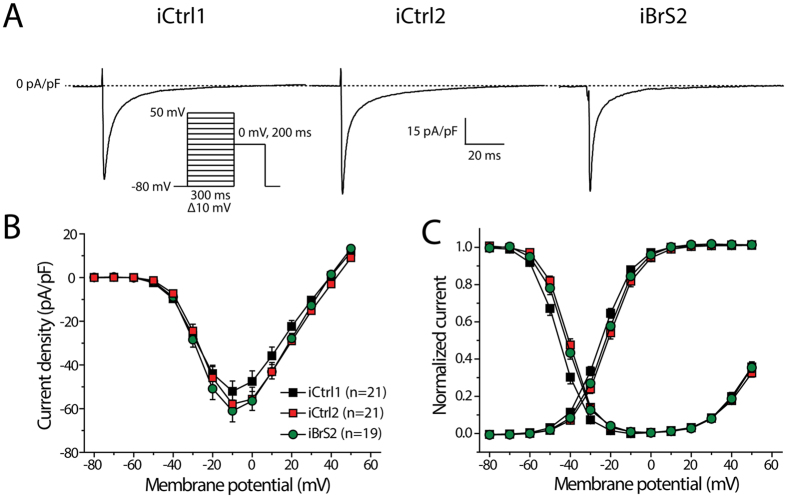
L-type calcium current (*I*_Ca,L_) in hiPSC-CMs from iCtrl1, iCtrl2 and iBrS2. (**A**) Typical traces of *I*_Ca,L_ measured at a test potential of 0 mV. Inset shows the used voltage clamp protocol. (**B**) I-V relationships of *I*_Ca,L_. (**C**) Voltage dependence of activation and inactivation.

Given the shorter APD that was found in iBrS2 hiPSC-CMs, *I*_Ca,L_ and *I*_to_ were measured in hiPSC-CMs from this cell line and compared with hiPSC-CMs from the two controls. Both these currents are implicated in BrS and could underlie the observed differences in APD. Fig. 4A shows typical traces of *I*_Ca,L_ measured at a test potential of 0 mV. *I*_Ca,L_ density was not affected in iBrS2 as compared to the two controls iCtrl1 and iCtrl2 (Fig. 4B). Also, voltage dependence of activation was not different between all groups, while iCtrl1 demonstrated a negative shift of 4 mV in voltage dependence of inactivation as compared to iCtrl2 (p < 0.05). Time constants of current inactivation, measured at a potential of 0 mV, were not statistically different between all groups (τ_fast_: 4.0 ± 0.2, 3.7 ± 0.2 and 3.6 ± 0.3 ms in iCtrl1, iCtrl2 and iBrS2, respectively; τ_slow_ 18.2 ± 0.8, 21.5 ± 1.3 and 21.5 ± 1.4 in iCtrl1, iCtrl2 and iBrS2, respectively).

**Figure 5:**
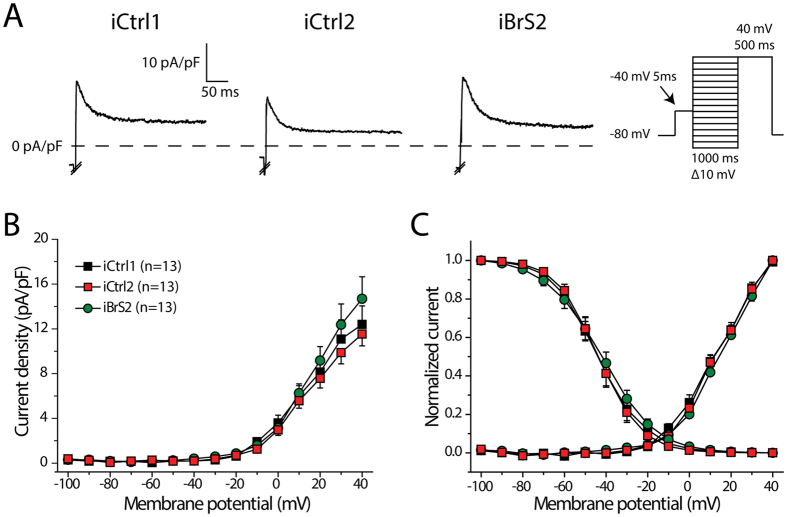
Transient outward potassium current (*I*_to_) in hiPSC-CMs from iCtrl1, iCtrl2 and iBrS2. (**A**) Representative trances of *I*_to_ elicited at a test potential of +40 mV. Inset shows the voltage clamp protocol, in which a prepulse of 5 ms to −40 is applied to activate and inactivate *I*_Na_. Please note that *I*_Na_ was cut-off in the depicted examples. (**B**) I-V relationships of *I*_to_. No statistical differences are present (One-Way Anova, p > 0.05) (**C**) Voltage dependence of activation and inactivation.

Figure 5A shows typical traces of *I*_to_ elicited at +40 mV. *I*_to_ was present in all cells measured and neither *I*_to_ densities (Fig. 5B) nor the voltage dependence of *I*_to_ (in)activation (Fig. 5C) were different between all groups. Moreover, there were no statistically significant differences in time constant of inactivation, as determined with a mono-exponential fit at a test potential of +40 mV. (τ: 28.1 ± 4.6, 26.0 ± 5.0 and 27.1 ± 3.3 ms in iCtrl1, iCtrl2 and iBrS2, respectively).

## Discussion

In this study we used hiPSC technology to generate cardiomyocytes from three patients with BrS that did not carry coding region mutations in the known BrS-associated genes. By comparing their electrophysiological properties to those of cardiomyocytes derived from two control individuals as well as an individual carrying the established mutation *SCN5A*-1795insD as positive control, we aimed to glean insight into the underlying mechanism of BrS.

Although the evidence supporting causality varies across genes, at least 20 different genes have so far been implicated in BrS^2^,^3^. They all encode ion channel subunits or proteins that putatively modulate ion channel function. In accordance, functional studies on mutations within these genes have consistently invoked abnormal ion channel function as a contributory mechanism, namely through a reduction in *I*_Na_ or *I*_Ca-L_, or through an increase in *I*_to_ and *I*_KATP_. As such, these observations point to a cellular electrophysiological defect in at least a subset of BrS patients. Around 80% of patients however do not carry coding region mutations in these genes^3^. In these patients, if a genetic component is operative, it is likely to involve three possibilities. It may entail (1) a non-coding genetic variant that alters the expression of the known BrS associated genes (and thereby acts through effects on the above-mentioned ionic currents), (2) mutations in genes encoding other proteins that affect the above-mentioned or other ionic currents, or alternatively, (3) mutations in genes that act through mechanisms that do not affect cardiomyocyte-intrinsic ion channel dysfunction.

Our electrophysiological studies in hiPSC-CMs from the three mutation-negative BrS patients did not uncover clear cellular electrophysiological differences compared to controls. As loss-of-function of *I*_Na_ is the most established underlying disease mechanism, we studied this current both directly by means of voltage clamp experiments, and indirectly, by assessment of the upstroke velocity of action potentials. Both methods did not reveal any abnormalities herein. In contrast, in the same experimental system we were able to detect loss of sodium channel function due to the *SCN5A*-1795insD mutation, a genetic defect that gives rise to an overlap phenotype of cardiac sodium channelopathy, encompassing BrS and cardiac conduction disease^19^. hiPSC-CMs carrying this variant (iSCN5A) displayed a clear reduction in *I*_Na_ density and a decreased AP upstroke velocity as reported previously^17^.

Apart from loss-of-function in *I*_Na_, loss- and gain-of-function in *I*_to_ and *I*_Ca,L_, respectively, have been proposed to contribute to BrS by loss of the AP dome epicardially^20^ or by affecting conduction^21^. One out of the three studied BrS cell lines (iBrS2) exhibited a slight reduction in APD, therefore we also studied *I*_to_ and *I*_Ca,L_ in this cell line. However, *I*_to_ and *I*_Ca,L_ in hiPSC-CMs from iBrS2 demonstrated similar densities and (in)activation properties as compared to the controls. Since we did not assess *I*_to_ and *I*_Ca,L_ in hiPSC-CMs from iBrS1 and iBrS3, we cannot completely rule out the possibility that these ion currents are affected in these groups. However, no differences in AP characteristics were observed, suggesting that no marked abnormalities in *I*_to_ and *I*_Ca,L_ are present that would contribute to the disease. It can be argued that contribution of *I*_to_ might be small in APs of hiPSC-CMs^22^; however, due to the *in silico* addition of *I*_K1_ the MDP in our measurements was negative to −80 mV, a potential were *I*_to_ is fully available (Fig. 5C). Our observations thus indicate cellular electrophysiological characteristics that do not differ from those of controls, thus supporting the hypothesis that the mechanism(s) underlying the phenotype in these patients may not involve cellular electrophysiological changes and is possibly not caused by mutations in ion channel genes or their modulatory subunits.

In the absence of distinct cellular electrophysiological changes, a number of processes may be hypothesized to contribute, solely or in aggregate, to BrS in these patients. A hypothesis that has increasingly gathered support concerns the role of fibrosis in the right ventricular myocardium^9^,^10^,^23^,^24^. This hypothesis is supported by the fact that BrS typically manifests in the fourth decade of life, which may represent an age at which the development of fibrosis becomes pathogenic. Another mechanism could entail decreased cardiomyocyte coupling through gap junctions^23^. Alternatively, non-genetic/environmental factors might play a more prominent role in these patients. Established modulators in this regard include drugs^25^, fever^26^ and vagal tone^27^. However, given the high prevalence of positive sodium channel blocker provocation tests in the relatives of the patients we studied (40–50% compared to 4.5% in the general population)^28^, a role for genetic factors seems likely. Lastly, BrS may be a multifactorial disorder, wherein multiple genetic and environmental factors each contribute to varying extent^29^. In such a scenario, small-effect defects in the ionic currents that shape the action potential might have been missed in the hiPSC-CM system we used.

Although our results indicate a lack of ion channel dysfunction in the three BrS patients, some considerations should be taken into account. It is well established that hiPSC-CMs exhibit electrophysiological characteristics that are comparable to fetal cardiomyocytes^30^. In our study, we partly corrected the electrophysiological immaturity by the *in silico* injection of *I*_K1_^31^, which consistently results in action potentials with a physiological resting membrane potential, thereby attaining physiological availability of *I*_Na_ and *I*_to_. However, important factors that modulate ion channel expression and function, such as beta subunits, transcription factors, microRNAs and epigenetic profile, may differ from mature cardiomyocytes^30^. Another feature of hiPSC-CMs is the lack of a defined intercalated disc, which in adult cardiomyocytes contains a distinct pool of sodium channels that have macromolecular interactions that differ compared to those at the lateral membrane^32^. Abnormalities in any of the proteins that are involved in the formation and maintenance of these distinct pools of sodium channels could be masked in hiPSC-CMs. In BrS, electrophysiological abnormalities including arrhythmia originate from the right ventricular outflow tract^8^,^33^. The transcriptome of cardiomyocytes from this region differs from that of other regions of the heart^34^ and this may confer different functional characteristics. The fate heterogeneity in cardiomyocyte populations derived by differentiation from hiPSC necessitates the use of specific culture protocols to enrich for specific cardiomyocyte subtypes. While differentiation protocols enriching for atrial-like cardiomyocytes^35^ and cardiomyocytes with properties of sinoatrial node cells^36^ have been developed, these are as yet not available for the right ventricular outflow tract. Notwithstanding, in our system we could recapitulate the effects of a BrS-causing *SCN5A* mutation.

In line with the cellular heterogeneity, we observed a high degree of variability in *I*_Na_, which could mask subtle differences in this current. However, one should note that AP upstroke velocities, reflecting sodium channel functionality, displayed less variation, possibly due to the selection of viable, regularly beating cells. The fact that no differences were observed in the latter parameter, which is more sensitive for the detection of small effects, supports our notion that *I*_Na_ is not affected in hiPSC-CMs from the selected BrS patients.

So far, electrophysiological studies in hiPSC-CMs have been mainly conducted in hiPSC-CMs derived from patients with known genetic defects and have mainly focused on the recapitulation of electrophysiological defects that had been previously described in other model systems such as heterologous expression systems or mouse models^17^,^37^,^38^. Our study for the first time attempts to glean insight into the mechanism of a cardiac disease in patients in which the underlying genetic defect is not known.

In conclusion, our study of hiPSC-CMs from three unrelated BrS patients without *SCN5A* mutations does not point towards an ion channel defect as a mechanism that contributes to the disease. This suggests that other pathophysiological mechanisms could be operative in these patients.

## Methods

### Genetic screening

The coding regions and splice sites of genes previously associated with BrS were screened for novel and rare variants in the 3 BrS patients by a next-generation sequencing (NGS) approach we used previously^39^. These genes were *SCN5A, SCN1B*, *SCN2B*, *SCN3B, SCN10A, CACNA1C*, *CACNB2*, *CACNA2D1, KCNH2*, *KCNE3*, *KCNE1L*, *KCND3*, *KCNJ8, ABCC9, TRPM4*, *HCN4, GPD1L*, *RANGRF*, *PKP2* and *FGF12*. The coding exons and flanking splice sites were captured from 200 ng of genomic DNA using the Agilent HaloPlex Target Enrichment system (Agilent Technologies) following the manufacturer’s protocol. 100-bp paired end read sequencing was conducted on a HiSeq sequencer (Illumina). Sequence reads were aligned to the human reference genome (standard 1000Genomes fasta GRCh37 alignment file) using BWA (version 0.6.2)^40^ and called using GATK (version 2.6)^41^ and SAMtools (version 0.1.19)^42^. On average, the mean depth was 503, and 96.4% of the targeted regions were covered at least 10 times. The Knime4Bio tool was used to manage and filter variants^43^. Synonymous variations that were not located at splice sites were excluded from further analysis. Heterozygous and homozygous coding and splice site variants with a minor allele frequency (MAF) below 1% in public databases (Exome Aggregation Consortium (ExAC), Cambridge, MA (URL: http://exac.broadinstitute.org), GoNL^44^ and the 69 genomes from Complete Genomics) were retained and validated by Sanger sequencing.

### Generation and characterization of hiPSC

The study was approved by the ethics committee of the Academic Medical Center of the University of Amsterdam (Netherlands) and conducted according to the approved guidelines. Informed consent was obtained from all study objects. The two control hiPSC lines (iCTRL1 and 2) and the hiPSC line carrying the *SCN5A*-1795insD mutation (i*SCN5A*) were generated and characterized previously^17^,^45^,^46^. Patient hiPSC lines (iBrS1-3) were generated from fibroblasts derived from skin punch biopsies. Dermal fibroblasts from the three patients were reprogrammed using the lentiviral STEMCCA system carrying the OCT4, KLF4, SOX2 and cMYC factors as previously described^45^. Pluripotency of the generated lines was confirmed by alkaline phosphatase staining, and by immunocytochemistry and RT-PCR for the detection of expression of the pluripotency markers OCT4, SOX2, NANOG, LIN28, SSEA-4, and TRA-1-60. The related primers, antibodies and applied protocols are described previously^45^. For each line, the potential to generate cell types derived from the three germ layers was tested by immunocytochemistry for the markers β-III-Tubulin, α-SMA and AFP after spontaneous differentiation of the hiPSC. Karyotype analysis was performed by the COBRA-FISH technique^47^.

### Differentiation of hiPSCs into cardiomyocytes and dissociation in single cells

All hiPSC lines were expanded as adherent cultures in feeder-free conditions on Matrigel-coated dishes in the presence of chemically defined medium (E8 Essential Medium, Life Technologies). Differentiation of hiPSC to cardiomyocytes (CMs) was performed over a period of 30 days following a previously reported protocol^48^ based on small molecules-mediated canonical Wnt pathway modulation (see Supplementary methods for more detail). Subsequently we enriched for CMs by switching the culture medium to DMEM supplemented with lactic acid (4 mmol/L) in substitution of glucose for 6 days as previously reported^49^. Cultures of hiPSC-CMs were then enzymatically dissociated into single cells using Elastase (Serva) and Liberase (Roche Chemicals) as described previously^31^. CMs were stained with anti-NKX-2.5 and cardiac TroponinT antibodies as described in the Supplementary methods. To allow for single-cell electrophysiological measurements, dissociated cells were plated at a low density on Matrigel-coated coverslips. Electrophysiological measurements were performed 9–14 days after dissociation.

### Electrophysiology

#### Data acquisition and analysis

Action potentials (APs) and *I*_Na_ were recorded using an Axopatch 200 B amplifier (Molecular Devices, Sunnyvale, CA, USA). Voltage control, data acquisition, and analysis were realized with custom software. Potentials were corrected for the calculated liquid junction potentials. Cell membrane capacitance (C_m_) was calculated by dividing the time constant of the decay of the capacitive transient after a −5 mV voltage step from −50 mV by the series resistance. Signals were low-pass-filtered with a cutoff of 5 kHz and digitized at 40 kHz for APs, 20 kHz for *I*_Na_, 10 kHz for *I*_Ca,L_ and 4 kHz for *I*_to_. For all lines and experiments, data was collected from at least 3 independent differentiations.

#### Action potentials

AP measurements were acquired at 36 ± 0.2 °C from single hiPSC-CMs using the amphotericin-B-perforated patch-clamp technique. Spontaneously beating hiPSC-CMS showing regular, synchronous contractions were selected. Pipettes (borosylicate glass) were filled with solution containing (in mmol/L): 125 K-gluconate, 20 KCl, 10 NaCl, 0.44 amphotericin-B, 10 HEPES; pH 7.2 (KOH). Bath solution contained (in mM): 140 NaCl, 5.4 KCl, 1.8 CaCl_2_, 1.0 MgCl_2_, 5.5 glucose, 5.0 HEPES; pH 7.4 (NaOH). Typically, hiPSC-CMs have a small or even complete lack of the inward rectifying potassium current (I_K1_)^31^. Consequently, hiPSC-CMs have a depolarized maximal diastolic potential (MDP) and are frequently spontaneously active^30^. To overcome these conditions, which limit the functional availability of *I*_Na_ and *I*_to_, we injected an *in silico I*_K1_ with kinetics of Kir_2.1_ channels through dynamic clamp, as previously described in detail^31^. In the present study, we consistently used an amount of 2 pA/pF peak outward current which resulted in quiescent hiPSC-CMs with a RMP of −80 mV or more negative that displayed ventricular-like APs in >90% of the cells (based on the proportion of cells with a plateau amplitude of >85 mV, measured 20 ms after the initiation of the AP upstroke; see Supplementary Fig. 7). The proportion of hiPSC-CMs displaying ventricular-like AP was not different between the groups. Because the vast majority of cells exhibited ventricular-like APs and a selection of hiPSC-CMs subtypes cannot be achieved during ion current measurements, we refrained from performing a selection based on action potential morphology. Action potentials were elicited at 0.5 to 3 Hz by 3-ms, ~1.2x threshold current pulses through the patch pipette. APs were characterized by MDP, maximum AP amplitude (APA_max_), AP duration at 20, 50 and 80% of repolarization (APD_20_, APD_50_, APD_80_ respectively), maximal upstroke velocity (V_max_) and plateau amplitude (APA_plateau_, measured 20 ms after the AP upstroke). Averages were taken from 10 consecutive APs and cells with an access resistance of >50 MΩ were excluded from analysis.

#### Membrane currents

*I*_Na_, *I*_Ca,L_ and *I*_to_ were measured using the ruptured patch-clamp technique with conventional voltage clamp protocols as depicted in the accompanying figures. *I*_Na_ was measured at room temperature, *I*_Ca,L_ and *I*_to_ were measured at 36 ± 0.2 °C. Cycle lengths were 5, 2 and 10 seconds for *I*_Na_, *I*_Ca,L_ and *I*_to_, respectively. *I*_Na_, *I*_Ca,L_ and *I*_to_ were defined as the difference between peak current and steady-state current. Current densities were calculated by dividing currents by C_M_. Steady-state activation and inactivation curves were fitted by using a Boltzmann equation: *I*/*I*_max_ = *A*/{1.0 + exp[(*V*_1/2_ − *V*)/*k*]}. In which *V*_1/2_ is half-maximum (in)activation potential and *k* is the slope factor. For voltage dependence of (in)activation of *I*_Na_, only cells with adequate voltage control were included, as ascertained by a slope factor >6 for both activation and inactivation. Time constants of inactivation of *I*_Na_ and *I*_Ca,L_ were determined by fitting a biexponential curve through the decay phase of the current using the equation: I/I_max_ = A_f_ × exp(−t/τ_f_) + A_s_ × exp(−t/τ_s_), in which A_f_ and A_s_ are the fractions of the fast and slow inactivation components, and τ_f_ and τ_s_ are the time constants of the fast and slow inactivating components, respectively. Time constants of inactivation of *I*_to_ was fitted with a mono-exponential equation. For *I*_Na_, pipette solutions contained (in mmol/L): 3.0 NaCl, 133 CsCl, 2.0 MgCl_2_, 2.0 Na_2_ATP, 2.0 TEACl, 10 EGTA, 5.0 HEPES; pH 7.2 (CsOH). Bath solution for *I*_Na_ contained (in mmol/L)): 20 NaCl, 120 CsCl, 1.8 CaCl_2_, 1.2 MgCl_2_, 11.0 glucose, 5.0 HEPES; nifedipine 0.01; pH 7.4 (CsOH). For *I*_Ca,L_ and *I*_to_, spontaneously beating cells were selected in the same bath solution as used for APs after which the solutions were switched to specific extracellular solutions. *I*_Ca,L_ was measured in a bath solution containing (in mmol/L): 145 TEA-Cl, 5.4 CsCl, 1.8 CaCl_2_, 1.0 MgCl_2_, 5.0 HEPES, pH 7.4 (NMDG-OH). For *I*_to_ measurements, bath solution, was the same as for APs, except that 0.5 mM CdCl_2_ was added to block *I*_Ca,L_ and reduce *I*_Na_. For *I*_Ca,L_ measurements, pipettes were filled with solutions containing (in mmol/L): 145 CsCl, 10 HEPES, 10 EGTA, 5 K_2_ATP, pH 7.2 (NMDG-OH). Pipette solution for *I*_to_ measurements contained (in mmol/L): 105 K-gluconate, 20 KCl, 5 NaCl, 1 MgCl_2_, 10 BAPTA (pre-dissolved in KOH), 5 MgATP, 10 HEPES, pH 7.2 (NMDG-OH).

### Statistics

Statistical analysis was performed with SPSS Statistics 22. Normality and equality of variance were tested by Shapiro-Wilk and Levene median test, respectively. For parameters with a normal distribution, One-Way ANOVA followed by Bonferroni *post hoc* tests were performed. In case of non-normally distributed parameters, Kruskal-Wallis tests followed by pairwise comparisons with Bonferroni error corrections were applied. In all tests, comparisons were done between the test group (i.e. iBrS1, iBrS2, iBrS3 or iSCN5A) and the two controls (i.e. iCtrl1 and iCtrl2). For frequency dependence among groups, Two-Way repeated measures ANOVA was used. Data are presented as mean ± SEM, unless stated otherwise. P < 0.05 defines statistical significance.

## Additional Information

**How to cite this article**: Veerman, C. C. *et al*. hiPSC-derived cardiomyocytes from Brugada Syndrome patients without identified mutations do not exhibit clear cellular electrophysiological abnormalities. *Sci. Rep.* **6**, 30967; doi: 10.1038/srep30967 (2016).

## Supplementary Material

Supplementary Information

## Figures and Tables

**Table 1:** Sodium current (*I*_Na_) density, voltage dependence of (in)activation and time dependence of inactivation from hiPSC-CMs from the two controls (iCtrl1 and iCtrl2), the three BrS patients (iBrS1, iBrS2, iBrS3) and from hiPSC-CMS carrying the mutation 1795insD in *SCN5A* (iSCN5A1795insD).

Group	Peak current density (pA/pF) (median ± IQR)	Voltage dependence of activation		Voltage dependence of inactivation		Time dependence of inactivation	
		V_1/2_ (mV)	κ (mV)	V_1/2_ (mV)	κ (mV)	τ_slow_ (ms)	τ_fast_ (ms)
**iCtrl1**	−93.6 ± 84.9 (n = 36)	−34.6 ± 0.5 (n = 26)	6.9 ± 0.1	−78.9 ± 1.4 (n = 15)*	−6.48 ± 0.2	5.2 ± 0.5 (n = 36)	1.50 ± 0.1
**iCtrl2**	−83.0 ± 83.3 (n = 31)	−33.0 ± 0.9 (n = 20)	7.0 ± 0.2	−85.0 ± 1.0 (n = 11)	−7.05 ± 0.3	5.9 ± 0.7 (n = 33)	1.50 ± 0.1
**iBrS1**	−81.7 ± 73.25 (n = 37)	−33.5 ± 0.5 (n = 25)	6.6 ± 0.1	−83.6 ± 0.9 (n = 15)	−6.6 ± 0.2	6.4 ± 0.7 (n = 37)	1.52 ± 0.1
**iBrS2**	−96.8 ± 70.5 (n = 28)	−34.7 ± 0.7 (n = 20)	7.0 ± 0.2	−84.5 ± 1.0 (n = 10)	−6.6 ± 0.2	7.2 ± 1.0 (n = 31)	1.55 ± 0.1
**iBrS3**	−151.7 ± 208.5 (n = 31)	−32.1 ± 0.6 (n = 29)	7.2 ± 0.2	−80.0 ± 0.9 (n = 13)†	−6.83 ± 0.2	7.3 ± 0.8 (n = 32)	1.68 ± 0.2
**iSCN5A**	−38.7 ± 55.03‡ (n = 25)	−31.8 ± 0.9 (n = 20)	6.7 ± 0.2	−83.7 ± 0.8 (n = 10)	−6.9 ± 0.2	16.5 ± 2.0 (n = 25)‡	2.55 ± 0.2‡

Data is presented as mean ± SEM, unless stated otherwise. V_1/2_, voltage of halfmaximum (in)activation; κ; slope factor of (in)activation; τ_fast_, fast time constant of inactivation; τ_slow_, slow time constant of inactivation.

^*^p < 0.05 vs. iCtrl2, iBrS1 and iBrS2 (One-way ANOVA, posthoc Bonferroni test).

^†^p < 0.05 vs. iCtrl2 (One-way ANOVA, posthoc Bonferroni test).

^‡^p < 0.01 vs. all groups (Kruskal-Wallis test, followed by pairwise comparisons).
