# Supplementary material for: hiPSC-derived cardiomyocytes from Brugada Syndrome patients without identified mutations do not exhibit clear cellular electrophysiological abnormalities
Source: Sci Rep. 2016 Aug 3;6:30967. doi: 10.1038/srep30967 (PMC4971529; doi:10.1038/srep30967)
Supplement: Supplementary Information [file srep30967-s1.pdf]

hiPSC-derived cardiomyocytes from Brugada Syndrome patients without identified mutations do not exhibit clear cellular electrophysiological abnormalities

Authors: Christiaan C. Veerman, MD #[1]; Isabella Mengarelli, PhD #[1]; Kaomei Guan, PhD [2]; Michael Stauske, MsC[2]; Julien Barc, PhD [1,3-5]; Hanno L. Tan, MD, PhD [1]; Arthur A.M. Wilde, MD, PhD [1]; Arie O. Verkerk PhD #[6]; Connie R. Bezzina, PhD#*[1]

SUPPLEMENTARY MATERIALS

Supplementary methods

Differentiation of hiPSC into cardiomyocytes

Differentiation of hiPSC to cardiomyocytes (CM) was performed by following a previously reported protocol based on small molecules-mediated canonical Wnt pathway modulation[1]. Briefly, differentiation was initiated by a 24h treatment with CHIR99021 (12uM, Selleckchem) followed by Wnt pathway inhibition mediated by IWP-4 (5uM, Stemgent) on days 4 and 5. The differentiating cells were cultured in RPMI medium supplemented with B27 without insulin for 7days and in presence of insulin from day 8 to day 30. Enrichment for CM was achieved by switching the culture medium to DMEM supplemented with lactic acid (4mM) in substitution of glucose for 6 day as previously reported[2].

Dissociation of hiPSC-CMs into single cells

hiPSC-CM-enriched cultures were dissociated following an protocol optimized from one previously described for isolation of rabbit sinoatrial node cells[3]. Specifically, patches of beating hiPSC-CM were mechanically transferred from the cultures to a low-Ca^{2+} Tyrode solution, containing (in mM): 140 NaCl, 5.4KCl, 0.01 $CaCl_2$, 1.0 $MgCl_2$, 5.5 glucose, 5.0 HEPES, and 14.1 creatine; pH 7.4 (NaOH) and incubated for 10 min at room temperature. Subsequently, Liberase (0.04mg/mL, Roche Chemicals) and Elastase (0.01mg/mL, Serva) were added and cells were incubated for 10 min at 37◦C while shaking gently. Supernatant was then removed and a Kraft–Brühe solution (37◦C) was added to stop the enzymatic dissociation. This Kraft–Brühe solution contained (in mM): 85 KCl, 30 K_2HPO_4, 5.0 $MgSO_4$, 5.5 glucose, 5.0 pyruvic acid, 5.0 creatine, 30 taurine, 5.0 Na-hydroxybutyricacid, 5.0 succinic acid, 2.0 Na_2ATP and 1% BSA;

pH7.2 (KOH). A short period of firm manual shaking followed by incubation at 37◦C for 10 min with shaking allowed for dissociation into single cells. The cells were then centrifuged and resuspended in basic differentiation medium, consisting of RPMI medium supplemented with B27, 20% FBS and penicillin (50U/mL)/streptomycin (50µg/mL). The cell suspension was subsequently plated on 0.1% gelatin-coated glass coverslips. Medium was replaced with serum-free medium after 24h and subsequently with antibiotic-free medium every 3–4 days. Electrophysiological analysis was performed 10–15 days after dissociation.

Immunofluorescence in hiPSC-derived CMs

hiPSC-CM were fixed with 3% paraformaldehyde, permeabilised with phosphate buffer saline (PBS)/ 0.3% Triton-X 100 (Sigma-Aldrich) and blocked in presence of 10% FBS/1% BSA in PBS. The samples were then incubated for 40 min at 37◦C with primary antibodies (NKX-2.5 antibody, rabbit polyclonal, SantaCruz Biotechnologies, and, cardiac TroponinT antibody, mouse IgG1, Thermo Scientific), washed in PBS and incubated with the appropriate Alexa Fluor 488- and Alexa Fluor 568-conjugated secondary antibodies for 40 min at room temperature. Nuclei were stained with 4', 6-Diamino-2-Phenylindole (DAPI, Invitrogen). Images were captured with an inverted microscope (Leica DMI 3000B) equipped with a DFC 345FX camera (Leica Microsystems).

Supplementary Figures

Supplementary figure 1

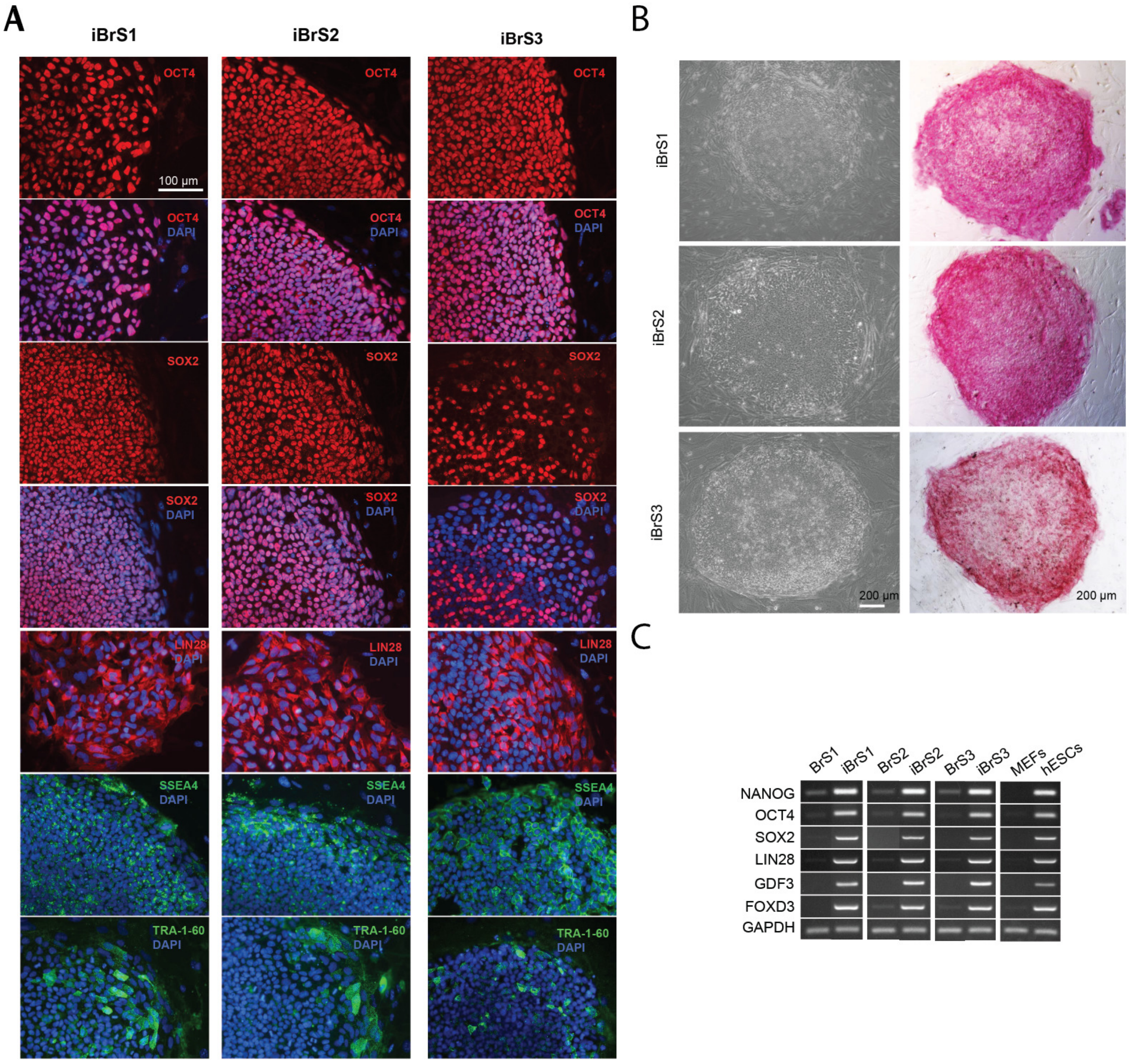

Supplementary figure 2

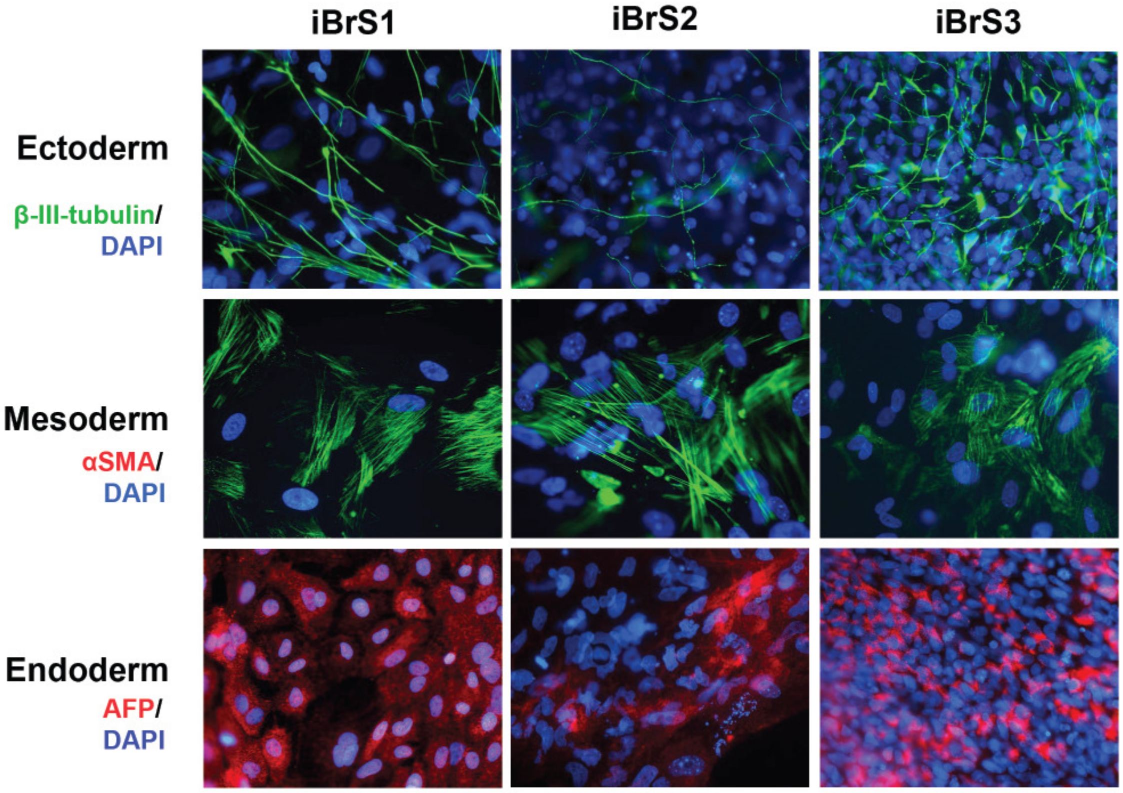

Supplementary figure 3

A) iCtrl1

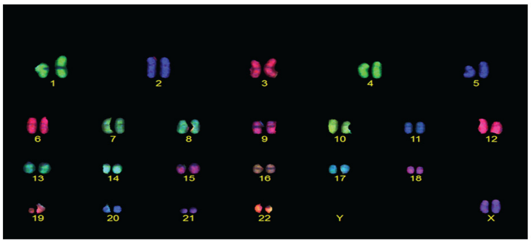

B) iCtrl2

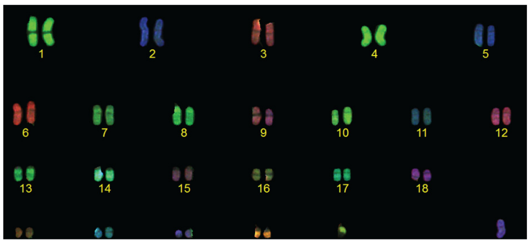

C) iBrS1

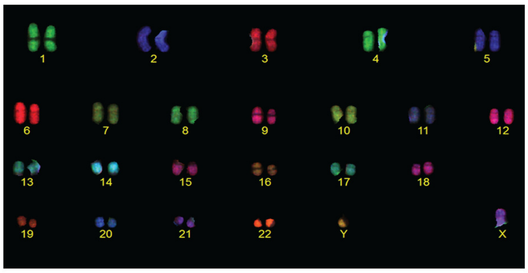

D) iBrS2

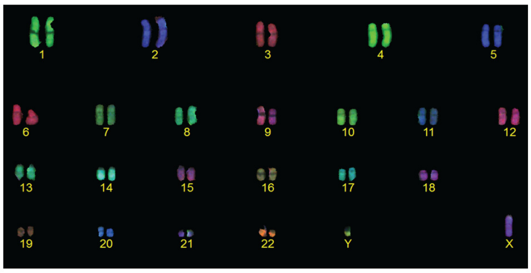

E) iBrs3

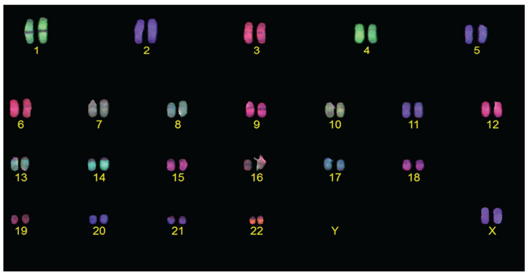

Supplementary figure 4

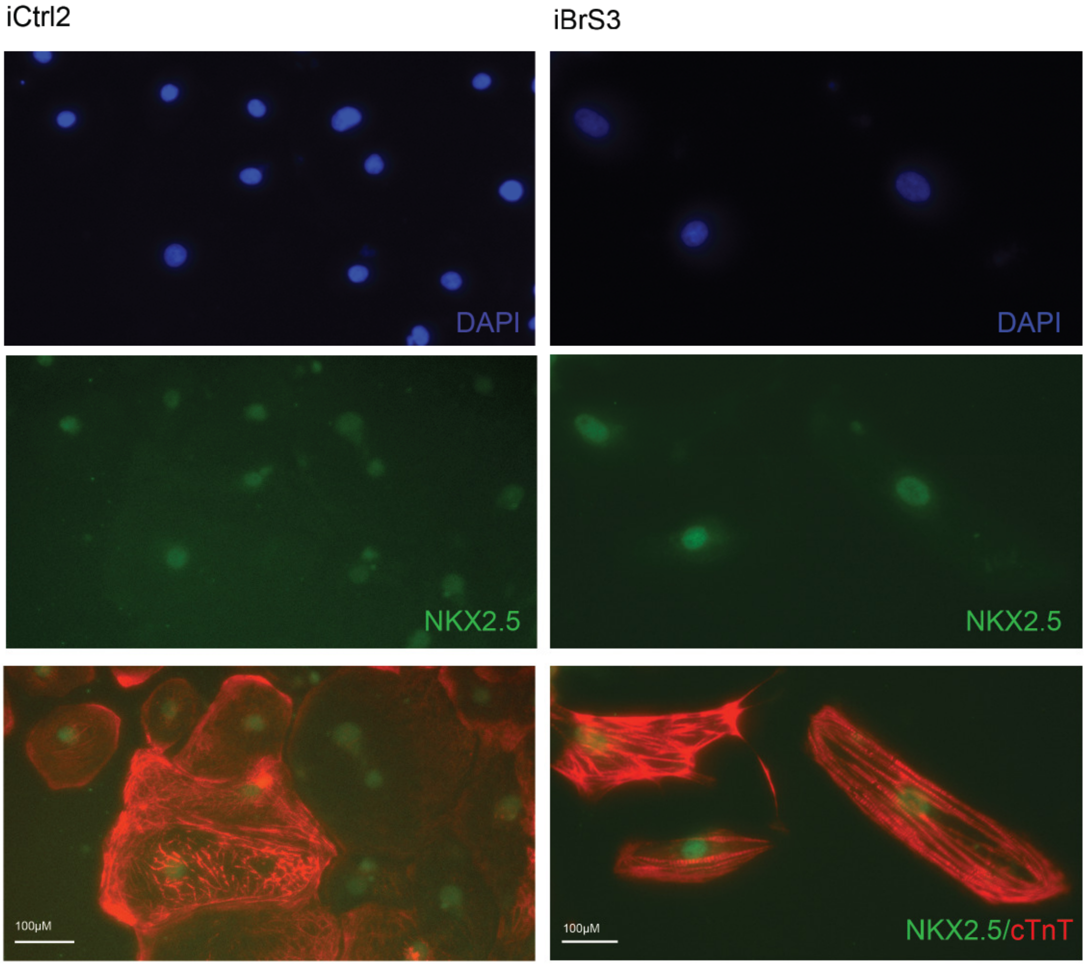

Supplementary figure 5

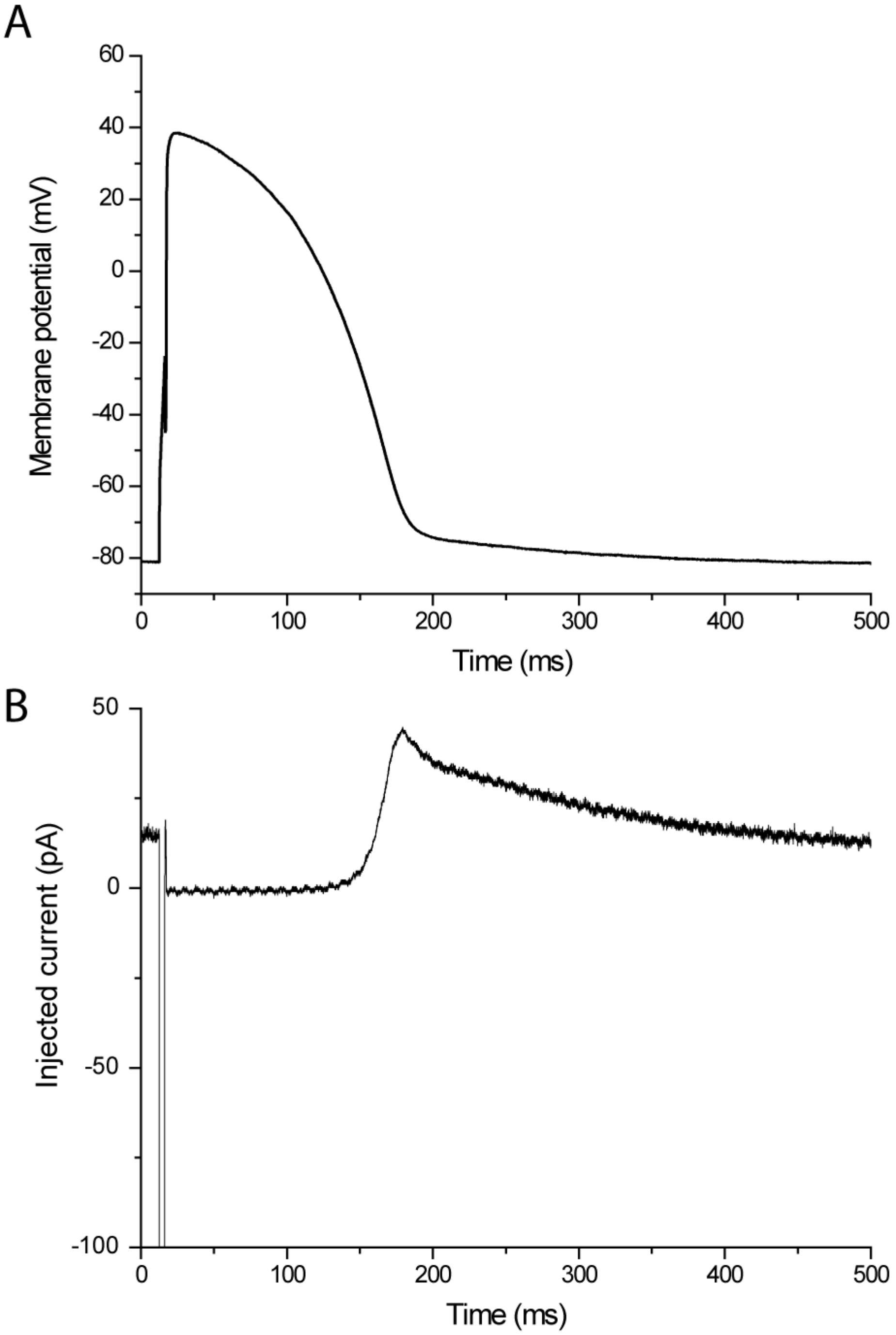

Supplementary figure 6

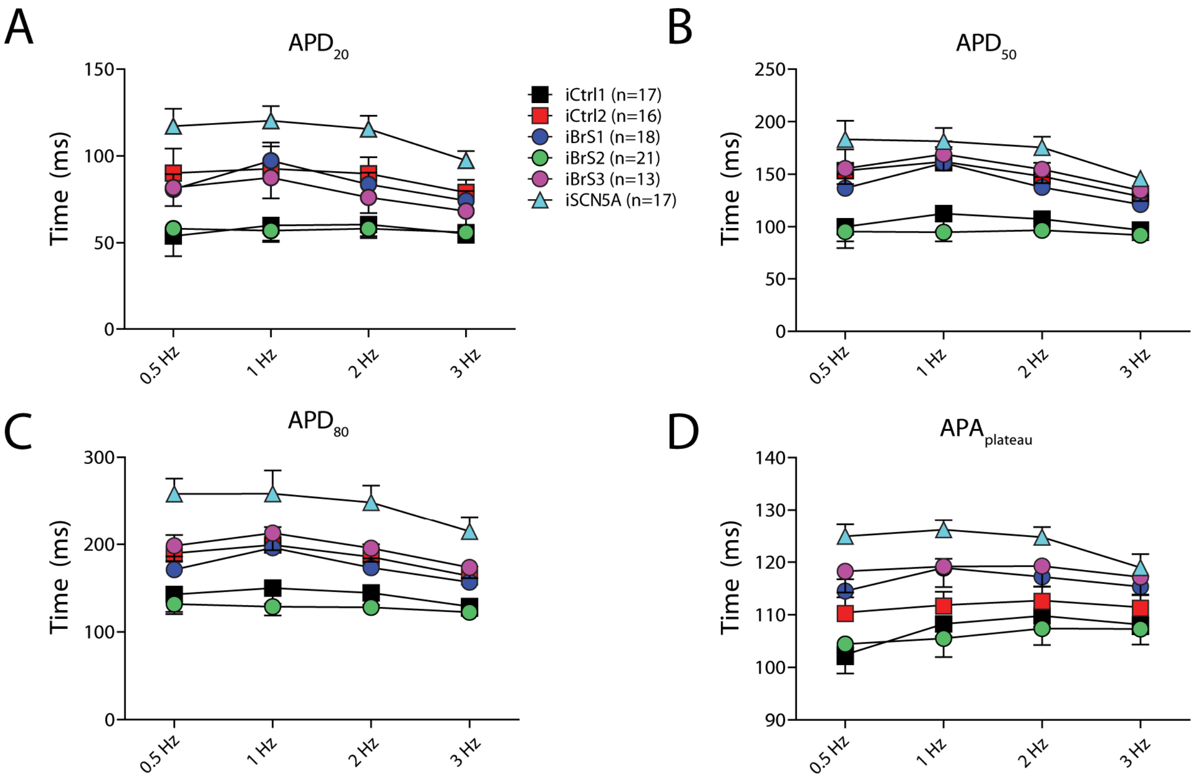

Supplementary figure 7

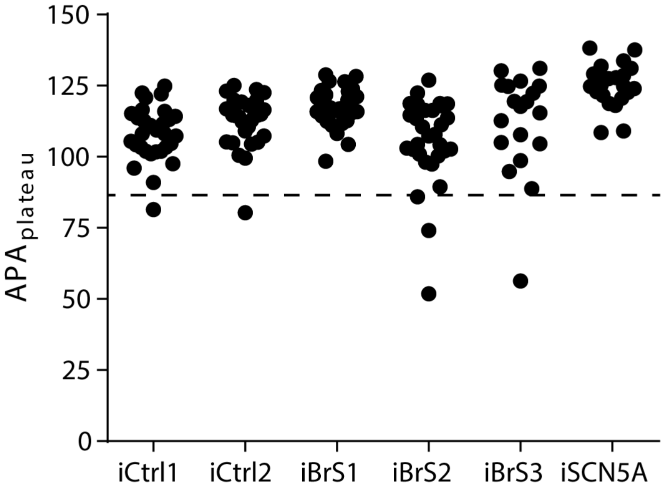

Supplementary figure legends

Figure S1 A. Immunofluorescence assay in undifferentiated BrS hiPSC lines showing protein expression of transcription factors (OCT4, SOX2, LIN28) and surface molecules (SSEA4, Tra-1-60) characteristic of the pluripotent state. **B.** Cytochemistry assay showing expression of the pluripotency marker human placental Alkaline Phosphatase in the three hiPSC lines derived from the Brugada patients **C** RT-PCR analysis of pluripotency markers expression in fibroblasts from BrS patients and the respective hiPSCs , demonstrating that the relevant pluripotency genes are expressed in the reprogrammed cells. Mouse embryonic fibroblasts (MEFs) and a human embryonic stem cells line (hESC) are shown as negative and positive controls, respectively.

Figure S2 Immunofluorescence detection of proteins, typically expressed in cell types derived from the three germ layers, in each of the hiPSC lines from Brugada syndrome patients(iBrS1, iBrS2, iBrS3) during differentiation. β III-tubulin, for ectoderm-derived neurons, α smooth muscle actin (αSMA) for a variety of mesoderm-derived cells, alpha feto protein (AFP) for endoderm-derived cells.

Figure S3 A-C. COBRA FISH-based karyotype analysis of undifferentiated hiPSC lines show normal chromosome sets for all hiPSC lines from Brugada patients.

Figure S4 Immunofluorescence detection of proteins typically expressed in cardiomyocytes (NKX2.5, cTNT) in representative cells derived from one control hiPSC-line (iCtrl2) and one Brugada syndrome (iBrS3) hiPSC line after application of the cardiomyocytes differentiation protocol**.**

Figure S5 A. Typical example of an action potential stimulated at 1 Hz upon *in silico* injection of I_{K1}. **B.** Corresponding computed I_{K1} current that is injected into the cells. A maximal peak outward current of 2 pA/pF is applied.

Figure S6

Average values of action potential duration at 20 (**A**), 50 (**B**) and 80% (**C**) of repolarization (APD_{20}, APD_{50}, APD_{80}, respectively) and action potential plateau amplitude ($APA_{plateau}$) (**D**) at a frequency range of 0.5-3 Hz. No additional effects were uncovered at different frequencies.

Figure S7

Dot plots of plateau amplitudes, measured 20 ms after initiation of the action potential upstroke. The dotted line indicates the cutoff value of 85 mV, distinguishing 'atrial'-like action potentials from 'ventricular'-like. This parameter was found to be highly discriminating in a previously published report, in which atrial-specific human embryonic stem cells derived-cardiomyocytes (hESC-CMs) were generated and characterized in detail[4]. Based on this parameter, the vast majority of cells generated with our differentiation protocol displayed a 'ventricular'-like action potential. The proportion of cells exhibiting 'ventricular'-like action potentials was not different across the groups (p=0.3, Fisher's exact test).

Supplementary References

1 Lian, X. *et al.* Directed cardiomyocyte differentiation from human pluripotent stem cells by modulating Wnt/beta-catenin signaling under fully defined conditions. *Nat Protoc* **8**, 162-175, (2013).

2 Tohyama, S. *et al.* Distinct metabolic flow enables large-scale purification of mouse and human pluripotent stem cell-derived cardiomyocytes. *Cell Stem Cell* **12**, 127-137, (2013).

3 Verkerk, A. O. *et al.* Dietary fish oil reduces pacemaker current and heart rate in rabbit. *Heart Rhythm* **6**, 1485-1492, (2009).

4 Devalla, H. D. *et al.* Atrial-like cardiomyocytes from human pluripotent stem cells are a robust preclinical model for assessing atrial-selective pharmacology. *EMBO Mol Med* **7**, 394-410, (2015).
